# A fluoroscopy-based planning and guidance software tool for minimally invasive hip refixation by cement injection

**DOI:** 10.1007/s11548-015-1252-8

**Published:** 2015-08-11

**Authors:** Daniel F. Malan, Stéfan J. van der Walt, Renata G. Raidou, Bas van den Berg, Berend C. Stoel, Charl P. Botha, Rob G. H. H. Nelissen, Edward R. Valstar

**Affiliations:** Department of Orthopaedics, Leiden University Medical Center, Albinusdreef 2, 2333 ZA Leiden, The Netherlands; Department of Intelligent Systems, Delft University of Technology, P.O. Box 5031, 2600 GA Delft, The Netherlands; Division of Applied Mathematics, University of Stellenbosch, Stellenbosch, 7602 South Africa; Department of Radiology, Leiden University Medical Center, Albinusdreef 2, 2333 ZA Leiden, The Netherlands; Department of Biomechanical Engineering, Delft University of Technology, Mekelweg 2, 2628 CD Delft, The Netherlands; Department of Biomedical Engineering, Technische Universiteit Eindhoven, 5600 MB Eindhoven, The Netherlands; Department of Orthopaedics, J11-R, Albinusdreef 2, 2333 ZA Leiden, The Netherlands

**Keywords:** Fluoroscopy, Digitally reconstructed radiograph, Pre-operative planning, Hip arthroplasty, Percutaneous, X-ray, Simulation

## Abstract

**Purpose:**

In orthopaedics, minimally invasive injection of bone cement is an established technique. We present HipRFX, a software tool for planning and guiding a cement injection procedure for stabilizing a loosening hip prosthesis. HipRFX works by analysing a pre-operative CT and intraoperative C-arm fluoroscopic images.

**Methods:**

HipRFX simulates the intraoperative fluoroscopic views that a surgeon would see on a display panel. Structures are rendered by modelling their X-ray attenuation. These are then compared to actual fluoroscopic images which allow cement volumes to be estimated. Five human cadaver legs were used to validate the software in conjunction with real percutaneous cement injection into artificially created periprothetic lesions.

**Results:**

Based on intraoperatively obtained fluoroscopic images, our software was able to estimate the cement volume that reached the pre-operatively planned targets. The actual median target lesion volume was 3.58 ml (range 3.17–4.64 ml). The median error in computed cement filling, as a percentage of target volume, was 5.3 % (range 2.2–14.8 %). Cement filling was between 17.6 and 55.4 % (median 51.8 %).

**Conclusions:**

As a proof of concept, HipRFX was capable of simulating intraoperative fluoroscopic C-arm images. Furthermore, it provided estimates of the fraction of injected cement deposited at its intended target location, as opposed to cement that leaked away. This level of knowledge is usually unavailable to the surgeon viewing a fluoroscopic image and may aid in evaluating the success of a percutaneous cement injection intervention.

## Introduction

The long-term survival of hip prostheses is primarily limited by the occurrence of aseptic loosening [1]. This pathological process involves extensive resorption of bone adjacent to the prosthesis and its replacement by fibrous tissue that offers little mechanical stability. Minimally invasive cement injection, already an established technique in the orthopaedic field of vertebroplasty [22], has in recent years been used experimentally to treat hip prosthesis loosening [8, 24].

Injected cement stabilizes a loosened prosthesis by re-establishing rigid mechanical contact between bone and the existing peri-prosthetic cement mantle [2]. Knowing the amount of cement that reaches its intended target may therefore be important in judging the success of a cement injection procedure.

Cement is injected through hollow needles that access the target lesions. Needle insertion may be guided by two-dimensional (2D) X-ray fluoroscopic supervision. Using intraoperative computed tomography (CT), more accurate guidance of needles’ out-of-plane displacement and rotation may be performed [23], but this presupposes the availability of CT hardware in the treatment room. More recently, systems have become available that use a combination of pre-operative CT and intraoperative fluoroscopy. One such example is XperGuide (Philips, Best, the Netherlands) [16, 25], where the accurate alignment of the patient, fluoroscopy images and the CT image volume depends on specialized hardware.

**Fig. 1:**
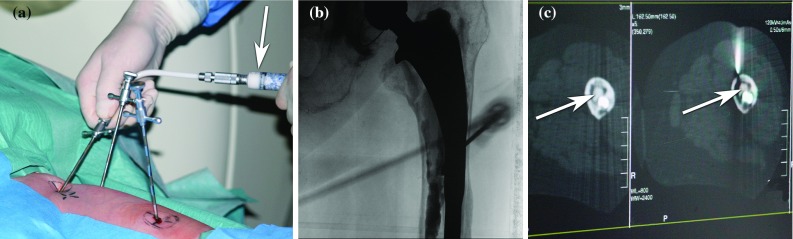
Percutaneous cement injection can be monitored by looking at **a** the syringe’s plunger, **b** fluoroscopic projections or **c** intraoperative cross-sectional CT slices

Once needles have been placed, cement is injected. During cement injection, the amount of deposited cement can directly be monitored by the position of the syringe’s plunger which is marked in millilitres, as shown in Fig. 1a. The spatial distribution of the injected cement can be estimated by looking at 2D X-ray fluoroscope projections as shown in Fig. 1b, where it appears as darkened image areas. The distribution of injected cement may also be monitored using three-dimensional (3D) intraoperative CT as shown in Fig. 1c. Currently, relying on 2D fluoroscopy for monitoring the flow of injected cement during minimally invasive prosthesis stabilization is the clinical standard [8, 24].

Cement injection is typically performed over the course of fewer than ten minutes due to polymethylmethacrylate (PMMA) bone cement hardening within this time span. Of the three intraoperative monitoring methods shown in Fig. 1, only CT provides true 3D information. CT cannot be continuously active throughout the procedure due to its higher radiation, as well as the need for the surgeon to have access to the patient. This makes 3D flow monitoring with CT scan difficult. Lastly, in practice, CT hardware is seldomly available in the treatment room. The patient needs to be slid into and out of the CT tunnel, and medical staff need to stand clear while the X-ray source is active to reduce radiation exposure. Three-dimensional CT can be performed intermittently, at best [7].

We present a proof-of-concept software tool that enables the volume of injected cement to be quantitatively estimated, using a single pre-operative CT image volume, a pre-operatively defined cement target, and one or more intraoperatively acquired 2D fluoroscopic images. This estimate may be performed for each image as it is read from the fluoroscope. The software is designed to augment specific aspects of a minimally invasive cement injection procedure including planning, execution and analysis.

In this paper, we describe our proof-of-concept planning software and then proceed to analyse its use in a pre-clinical cadaver experiment that was performed at our institution. In this experiment, percutaneous cement injection was planned in HipRFX and subsequently performed as for real human patients by an experienced orthopaedic surgeon.

## Materials and methods

### Workflow

**Fig. 2:**
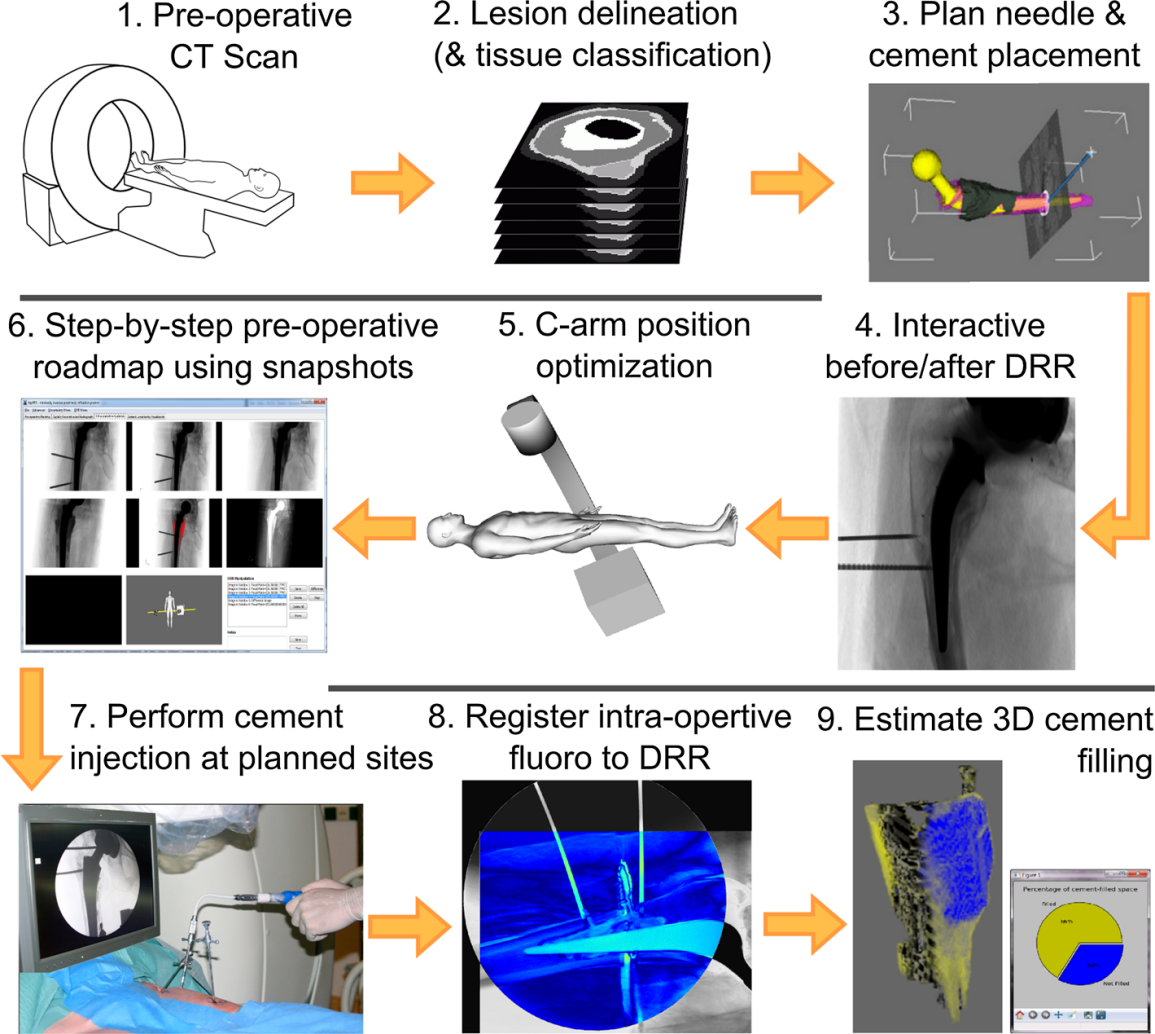
The envisaged workflow of planning and performing a minimally invasive hip refixation. Our proof-of-concept HipRFX software currently performs steps *3*–*4* and *9*. Steps *8* and *9* could conceptually be performed continually, in real time, during the execution of step *7*

The workflow in which our software is to be used is illustrated in Fig. 2. Our proof-of-concept software performs steps 3–4 and 9. Steps 2 and 8 are currently performed with human intervention, using external software.

Firstly, our software allows a surgeon to plan a cement injection procedure using a pre-operatively acquired CT as template. Secondly, our software can compute digitally reconstructed radiographs (DRRs) from a pre-operative CT [11]. DRRs are simulated 2D X-ray radiographs, computed from CT. DRRs can be used in guidance, or in estimating cement quantities. Thirdly, our software allows for a rough quantitative estimate of the resulting 3D cement distribution to be made. This is done by comparing the observed intraoperative fluoroscopic images to DRRs that are generated from the pre-operative CT volume.

When using HipRFX, the first step is to load a pre-operative CT volume of the affected hip. The user then specifies a 3D target “mask” that delineates the sub-volume(s) that should be filled with bone cement, i.e. osteolytic lesion(s). In our experiments we used the stand-alone MITK software [20] to perform these segmentations, but any medical volume segmentation tool may be used for this purpose. The segmented cement mask is then read by HipRFX as a binary image volume input file.

Once cement injection targets have been defined, the user has the possibility of adding and positioning virtual cement injection needles that access the designated target(s). If desired, additional segmented anatomical structures may be concurrently loaded and displayed.

From the moment that a CT volume is loaded, our software is able to interactively simulate fluoroscopic images based on a virtual C-arm that may be freely rotated. The goal of this simulation is to subsequently compare it with the actual intraoperative fluoroscopic images. Images may be simulated with or without designated needles and injected cement. By comparing actual intraoperative fluoroscopic images to these simulated images—both in the complete absence and in the complete presence of the intended cement filling—we generate 2D difference images between observed and simulated outcomes. These difference images are transformed to a 2D “cement filling” images that may be back-projected into the 3D volume to create a volumetric cement filling map. The algorithm’s sensitivity to image noise may be determined for each position in the cement filling image. Noise has the highest impact in regions where the image intensity difference between a low and high cement filling is small. This is further described in “Sensitivity of the output to image noise” section.

### Software user interface

HipRFX is implemented as a module in the DeVIDE Runtime Environment [5] and makes extensive use of the Visualization ToolKit (VTK), NumPy and SciPy [27].

**Fig. 3:**
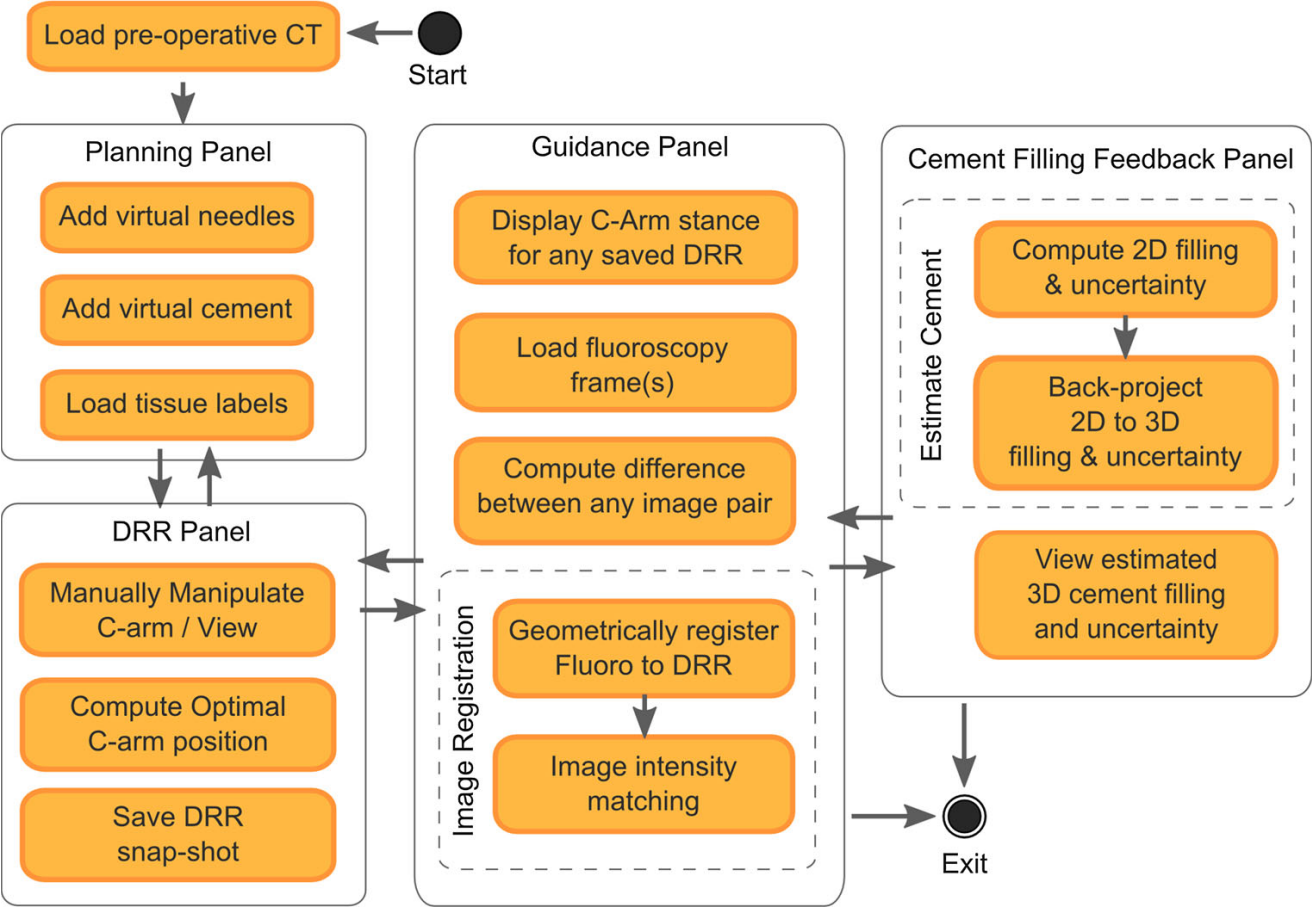
Overview of HipRFX’s main activities, split among its four panels. Components are grouped as functional units

The functionality contained in HipRFX is split across four separate panels, as explained in Fig. 3. Each panel has its own distinct purpose. We now describe the functionality and underlying methods of each panel separately. In its current proof-of-concept form, the image registration and intensity matching is performed by an external tool we built with scikit-image [28].

#### Planning panel

**Fig. 4:**
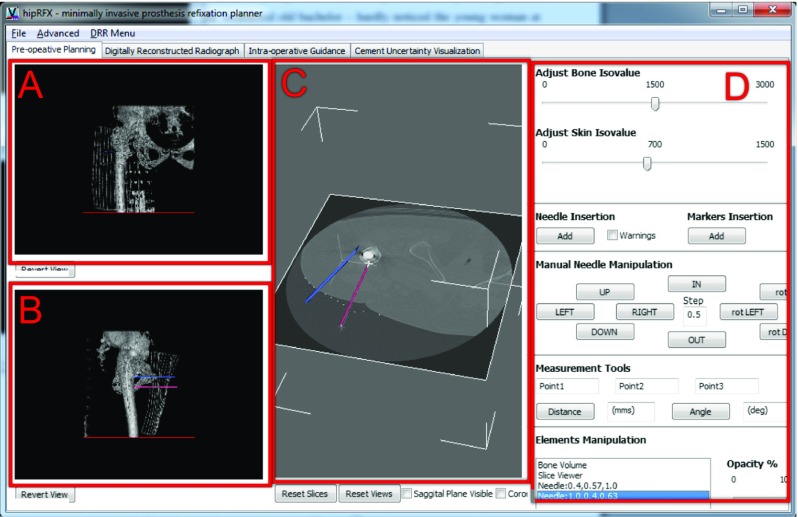
The planning panel provides 3D context renderings (*A*, *B*), an interactive slice view, and control buttons (*D*). Cement injection needles (shown here in *blue* and *red*) can be added to reach desired cement injection targets

The first view the user is presented with is the planning panel, shown in Fig. 4. As soon as a CT volume is loaded, it is rendered in a slice view and as a 3D volume. The panel contains two viewports showing 3D renderings of the hip’s bony structures from anterior-posterior (A) and medio-lateral (B) perspectives. To facilitate the positioning of the needles, the isovalues used for 3D rendering may be adjusted to provide the right amount of context. The central viewport contains a slice-based viewer that can be manipulated interactively (C). The user may load 3D segmented structures of interest. Examples include osteolytic lesions, or arteries and nerves that need to be avoided during surgery. Any number of cement injection needles can be virtually inserted into the CT volume and manipulated. The right-hand panel (D) contains controls to adjust the viewports’ display parameters, to perform distance and angle measurements, and to add, manipulate and remove needles.

**Fig. 5:**
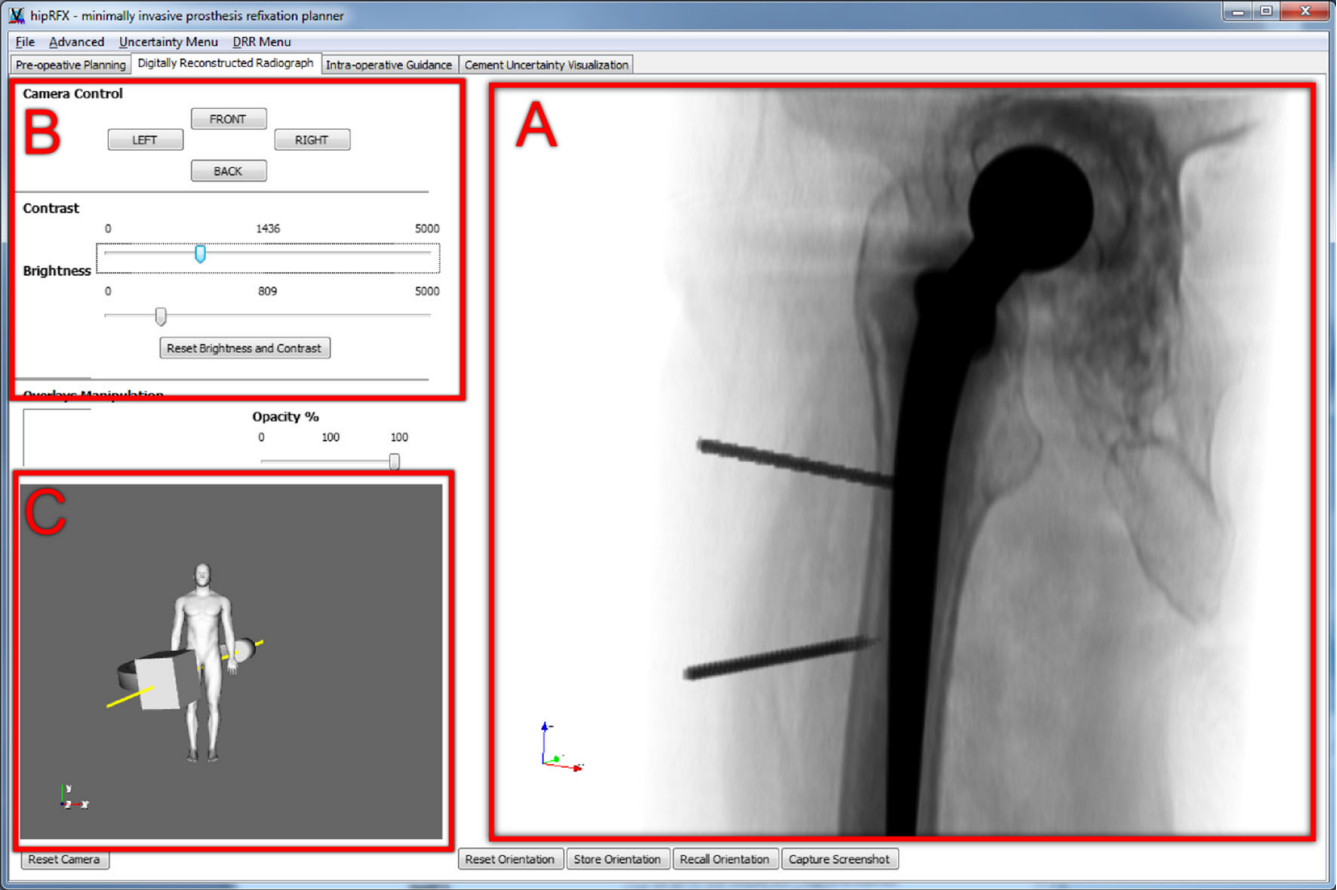
The DRR panel interactively simulates the fluoroscopic view a surgeon would see (*A*), including the effect of adding needles or bone cement. Snapshots may be saved at the press of a button, for later reference

**Fig. 6:**
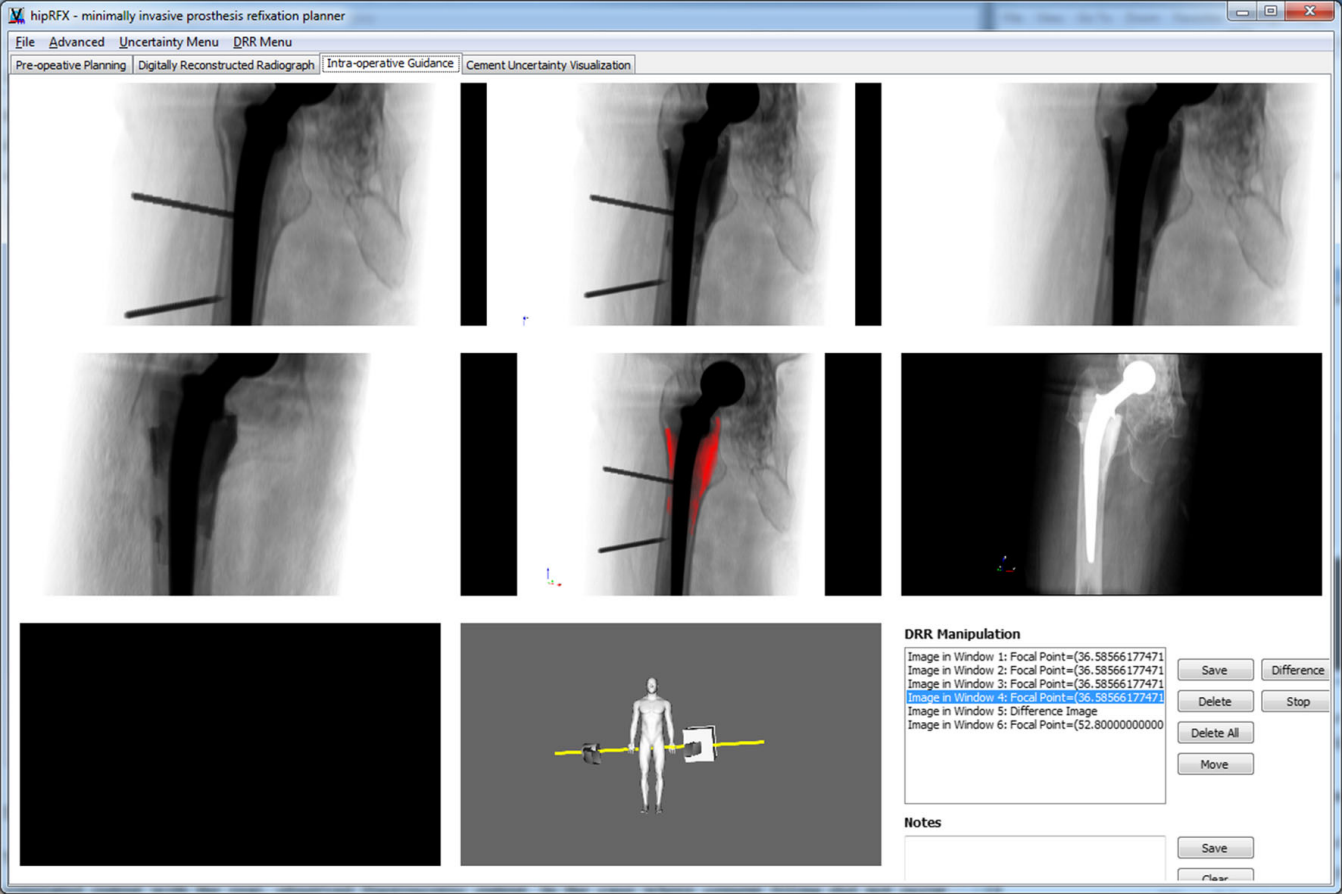
The guidance panel records simulated fluoroscopic snapshots. Differences between frames may be computed and displayed as overlays (shown in *red* in the *centre* snapshot). An example of an X-ray-mode DRR is shown in the *centre right*. In the *centre* of the *bottom row*, the fluoroscope C-arm’s position is shown that corresponds to the selected snapshot

#### DRR panel

The digitally reconstructed radiograph (DRR) panel shown in Fig. 5 allows a simulated fluoroscope to be interactively viewed and manipulated. The majority of the panel is taken up by the interactively rendered fluoroscopic image (A). Each image frame is created by a ray casting algorithm that directly simulates the propagation of X-rays through the patient [4]. The user may manually adjust the brightness and contrast to match the operating room fluoroscope’s settings (B), either to predetermined values obtained from calibration or visually.

An interactive 3D representation of the patient and the fluoroscopic C-arm is provided (C). The orientation of the C-arm and accompanying fluoroscopic view may be interactively manipulated to match the operating room’s set-up.

The image may be inverted to either emulate fluoroscopy or X-ray radiographs—the simulated X-ray attenuation algorithm is identical between these modes. This is an aesthetic choice to be made by the modality that the surgeon is most familiar with.

Needles or segmented cement targets that have been loaded in the planning view (Fig. 4) are realistically overlayed in the DRR image. In this way, the operator sees a simulation that corresponds to the fluoroscopic view he/she would see intraoperatively.

Snapshots can be saved to the step-by-step guidance panel described in the next subsection. With each snapshot, the C-arm orientation is stored as well. Snapshots may be used as a road map to guide the surgeon along planned steps during the execution of a minimally invasive procedure.

#### Step-by-step guidance panel

In the DRR panel shown in Fig. 5, the operator is allowed to store a number of DRR snapshots for later reference. Snapshots are displayed in a grid view, and clicking on any snapshot recalls the corresponding C-arm orientation that was used. These snapshots can act as an intraoperative road map since at any time, the operator may visually compare them to the live fluoroscopy image.

Changes in fluoroscopic images may be subtle between steps, e.g. between partial and complete cement filling. The software can highlight the difference between any pair of snapshots—this creates a copy of the image with differences overlayed in blinking red (see Fig. 6).

#### Cement filling feedback panel

**Fig. 7:**
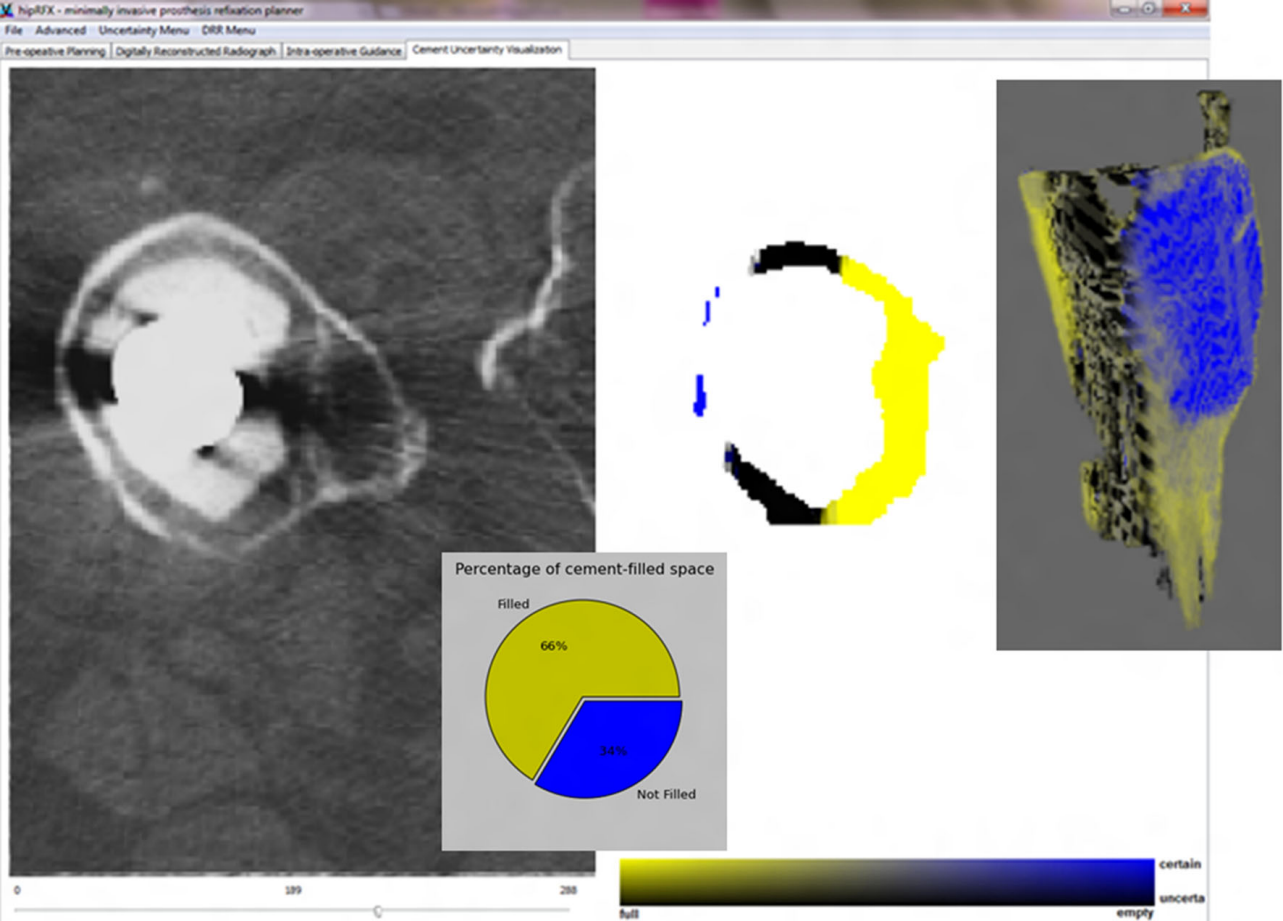
The cement filling feedback panel. A slice through the CT volume is shown on the *left*, with a 3D rendering of the whole cement target on the *right*. *Yellow* indicates target areas that are filled with cement, whereas *blue areas* indicate a lack thereof. Luminance increases with certainty

Differences between real intraoperative fluoroscopic images and DRRs may be analysed to yield estimates of cement filling. Along with filling estimates, sensitivity to image noise, i.e. “certainty”, may be computed. We discuss the way in which this “filling certainty ” is implemented in “Sensitivity of the output to image noise” section. The purpose of the cement filling feedback panel is to visualize these computed values. Using a bivariate colour map similar to those of Moreland [21], we represent certainty with luminance and filling with hue—see Fig. 7.

### Algorithm

**Fig. 8:**
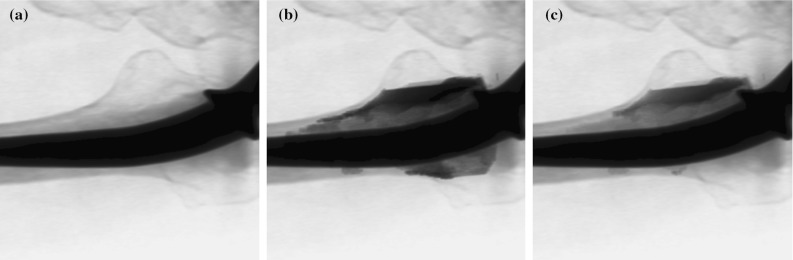
**a** Pre-operative DRR showing no injected cement. **b** Post-operative DRR showing complete cement filling of the intended cement target. **c** Intraoperative fluoroscopic image showing partial cement filling

We are able to estimate the volume of injected cement by comparing an observed fluoroscopic image to simulated fluoroscopic images. First, we generate a set of two DRRs. The first DRR simulates the fluoroscopic view in the absence of percutaneously injected cement. The second DRR simulates the fluoroscopic view when the target is completely filled with cement. During the cement injection procedure, real observed fluoroscopic images represent scenarios that lie in between these two extremes. An example of these inputs to our algorithm is shown in Fig. 8.

By numerically computing the image intensity differences between the observed fluoroscopic image and the two DRRs, our software numerically estimates the amount of cement that was injected.

#### Computing the amount of cement

**Fig. 9:**
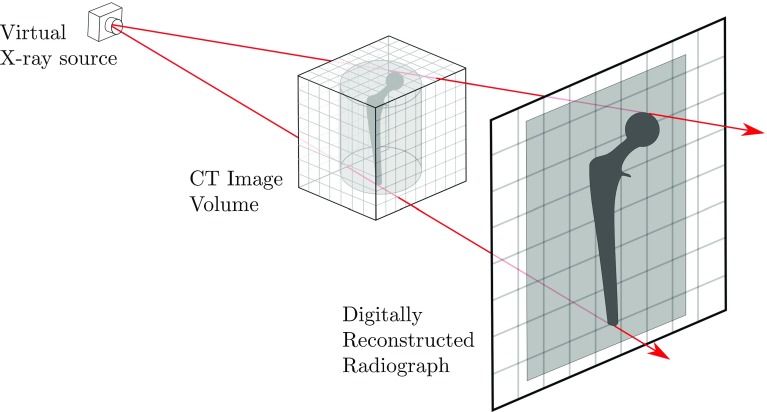
DRRs are formed by simulating the propagation of X-rays through a CT image volume

The DRR image formation process is illustrated in Fig. 9. Every element in the CT volume has at least one X-ray path that passes through it to the DRR image plane. The DRR formation is a discrete approximation of the physical X-ray imaging process.

**Fig. 10:**
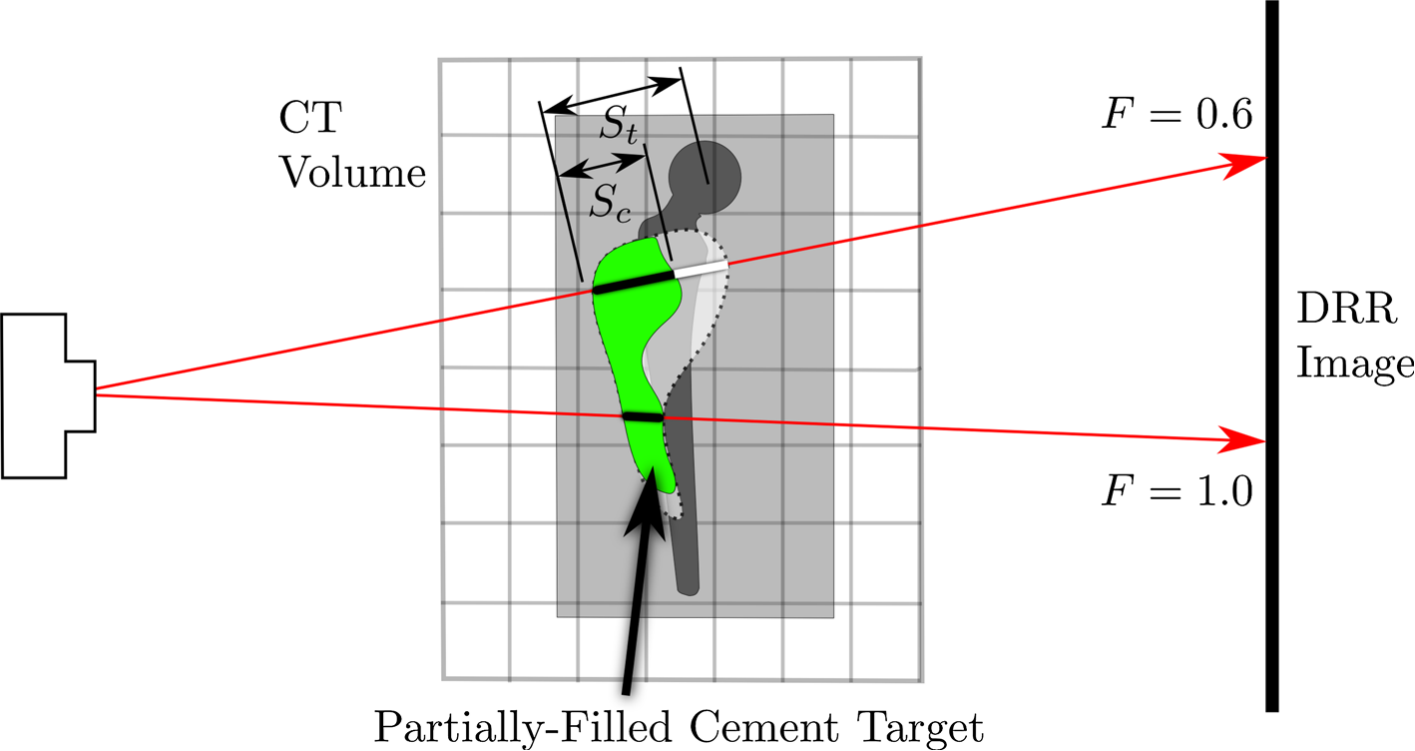
The filling fraction $F=s_\mathrm{c}/s_\mathrm{t}$ is the distance that each ray passes through cement, expressed as a fraction of the target area’s thickness along that ray

We can estimate the total volume of cement in the 3D CT by estimating the distance that each ray travelled through cement on its path from X-ray source to the image. The distance that each ray travelled through cement can be expressed as the distance that it travelled through the target, multiplied by the fraction of this distance that was filled with cement. We call this fraction the “fill fraction”, $F=s_\mathrm{c}/s_\mathrm{t}$ as shown in Fig. 10.

An X-ray beam’s flux exponentially decays as it passes through a uniform solid [14]. There exists a linear relationship between the length of an X-ray path through a uniform substance and the cumulative exponential absorption coefficient $\mu _\mathrm{ray}$ by which the ray is attenuated. Using similar notation as in Fig. 10, we rewrite the fill fraction as $F=\mu _\mathrm{c}/\mu _\mathrm{t}$. Here $\mu _\mathrm{c}$ is the cumulative X-ray attenuation coefficient contributed by the cement-filled portion of the target and $\mu _\mathrm{t}$ that which the target would have contributed if it was completely filled with cement. The superposition principle [13] allows us to express $\mu _\mathrm{c}$ and $\mu _\mathrm{t}$ each as the cumulative attenuation coefficient of the entire ray from source to image plane minus the portion that passes through no cement. We therefore rewrite the fill fraction as

1 $$\begin{aligned} F=\frac{\mu _\mathrm{partial}-\mu _\mathrm{none}}{\mu _\mathrm{full}-\mu _\mathrm{none}}. \end{aligned}$$

The subscript “none” refers to the entire ray where cement is completely absent—this represents the pre-operative scenario where no cement has yet been injected. “Partial” refers to the entire ray passing through the partially filled volume—this represents a typical intraoperative scenario. “Full” refers to the hypothetical ideal case where complete cement filling of the target is achieved.

By taking the response curve of the X-ray detector into account [6], one can show that the logarithm of the image brightness at any position in the fluoroscope’s image plane is linearly proportional to the accumulated X-ray attenuation along the path of the X-ray terminating at that point. Thus $\log (I)\approx -k\cdot \mu _\mathrm{ray},$ where *k* is some constant and *I* is the image intensity expressed as a value between 0 and 1. This allows us to rewrite Eq. 1 in terms of image intensities:

$$\begin{aligned} F=\frac{-\log \left( I_\mathrm{partial}\right) +\log \left( I_\mathrm{none}\right) }{-\log \left( I_\mathrm{full}\right) +\log \left( I_\mathrm{none}\right) } \end{aligned}$$

which becomes

2 $$\begin{aligned} F=\frac{\log \left( I_\mathrm{partial}/I_\mathrm{none}\right) }{\log \left( I_\mathrm{full}/I_\mathrm{none}\right) }. \end{aligned}$$

$I_\mathrm{none}$ and $I_\mathrm{full}$ refer to the fluoroscopy images seen when either no cement is injected and when the target volume is completely cement-filled. In an intraoperative setting, one never has access to $I_\mathrm{full}$ while $I_\mathrm{none}$ could be recorded at the start of the procedure. We chose to simulate both $I_\mathrm{none}$ and $I_\mathrm{full}$ by using our DRR algorithm on the pre-operative CT volume. Note that $I_\mathrm{partial}$ corresponds to the observed intraoperative fluoroscopy image that is available continuously during the cement injection procedure. An example of $I_\mathrm{none}$, $I_\mathrm{full}$ and $I_\mathrm{partial}$ is shown in Fig. 8.

**Fig. 11:**
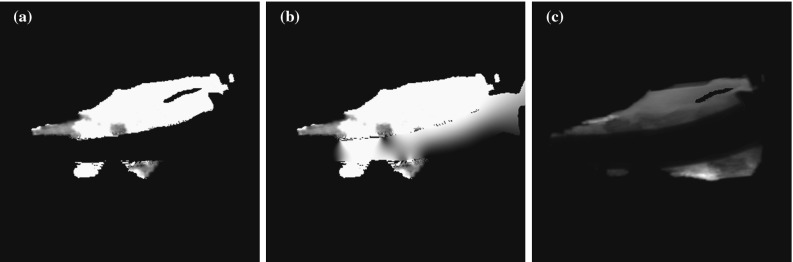
**a** The cement filling image *F* from Eq. 2. **b** *F*, after its interpolation to the area spanned by $I_\mathrm{cement}$. **c** The certainty image *C* computed using Eq. 4

This triad of images allows us to compute *F* for all pixels in the 2D fluoroscopy image where these values are defined, as shown in Fig. 11. The value of *F* for an arbitrary pixel in the image is denoted by $F_{x,y}$.

**Fig. 12:**
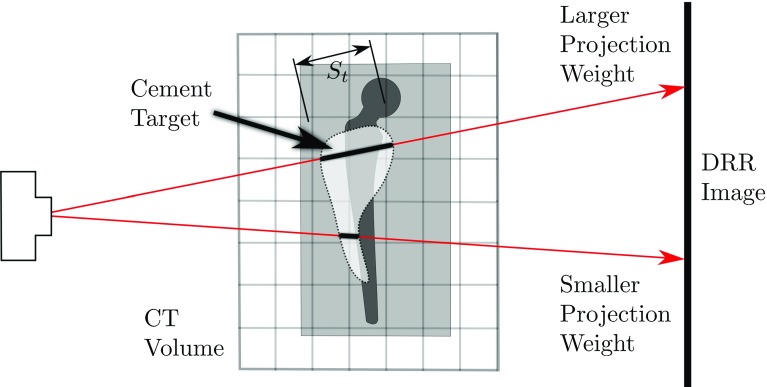
The projection weights scale linearly with the thickness of the cement through which rays pass to reach the image plane

To obtain a quantitative cement volume estimate, we need to weigh each pixel’s $F_{x,y}$ with the thickness $s_{t}$ of the cement target at the point through which the associated ray passed. We call these the “projection weights”—illustrated in Fig. 12.

**Fig. 13:**
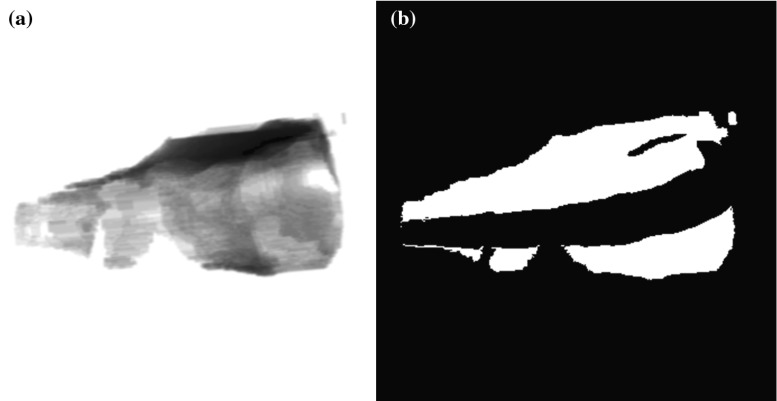
**a** The “cement-only” image $I_\mathrm{cement}$. **b** The subset of the image over which *F* can be computed using Eq. 2

The projection weights correspond directly to $s_{t}$ in Fig. 10, albeit appropriately scaled to yield a millilitre-valued output. The projection weights *W* are directly proportional to the attenuation coefficients that a completely cement-filled target would contribute to the rays terminating on each pixel. This is illustrated in Fig. 12. $I_\mathrm{cement}$ is the image that would result from X-rays passing only through the completely filled cement target and nothing else, as shown in Fig. 13.

**Fig. 14:**
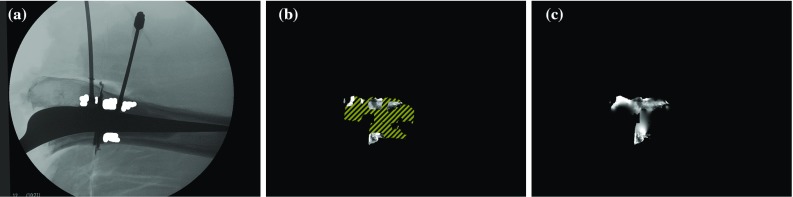
A mask was used to designate a valid subset of the computed cement filling projection in our experimental data. **a** Manually selected regions are superimposed in *white* and exclude confounding objects such as metal needles. **b** Input to the interpolation algorithm. Non-computable regions are shown with a diagonally hatched pattern. **c** The interpolated output

Mathematically:

$$\begin{aligned} W\propto s_\mathrm{t}\propto \mu _\mathrm{t}=-\frac{1}{k}\log I_\mathrm{cement}. \end{aligned}$$

As $F=s_\mathrm{c}/s_\mathrm{t}$, it follows that $W\cdot F\propto s_\mathrm{c}$. Looking back at Fig. 10, we note that summing $s_\mathrm{c}$ for every ray through the CT volume provides an approximation to the amount of cement contained in the partially filled target.

We can now compute an absolute quantitative estimate of the volume of the filled portion of the cement target. This is done by calculating the weighted sum of the fill fractions over all image pixels and multiplying the result with the volume of the complete cement target, in millilitres:

3 $$\begin{aligned} V_\mathrm{filled}=\frac{\sum _{x,y}\left( W_{x,y}\cdot F_{x,y}\right) }{\sum _{x,y}W_{x,y}}\cdot V_\mathrm{target}. \end{aligned}$$

A necessary prerequisite for computing Eqs. 2 and 3 is that all relevant DRR and fluoroscopy images are registered, i.e. that their field of view are the same, that they have the same brightness and contrast and that their subject is in the same position. In our proof-of-concept system and in our experimental set-up, calibration was approximated visually. In a practical clinical system, such pre-operative registration should instead be automatically and robustly implemented, and orientation recorded via angle encoders attached to the imaging hardware. To correct for mismatches in our experiments, we applied suitable rigid similarity transformation that corrects for scale, rotation and translation in each fluoroscopic image. These parameters were estimated from four manually selected point correspondences in each image pair. Brightness and contrast were corrected by linearly adjusting the intensity of the fluoroscopic image to match that of the DRR in a least squares sense.

Additional caveats apply. The cement fill fraction *F* in Eq. 2 is only defined in image regions onto which the cement target project, and only where the image brightness is not fully saturated. This is illustrated in Fig. 13.

All other areas in the image are marked as non-computable. Non-computable areas include those where the thick metal prosthesis completely absorbs the X-ray beam, as this would result in a zero-valued denominator $I_\mathrm{none}$ in Eq. 2. In non-computable areas falling inside the domain of $I_\mathrm{cement}$, *F* is interpolated using thin plate spline radial basis functions, as in Figs. 11 and 14. In our experiments, we manually delineated suitable regions within the domain of $I_\mathrm{cement}$ to serve as input to the interpolation algorithm, thereby excluding areas where needles were present or where cement leaked into the incision created during preparation of the test femurs. This is shown in Fig. 14.

Once *F* has been computed over the whole 2D projected cement filling domain, it may be back-projected along the ray path to 3D and visualized in the cement filling feedback panel as shown in Fig. 7.

#### Sensitivity of the output to image noise

Fluoroscopy images contain some degree of image noise that may distort the derived cement filling values. In areas occluded by radio-dense materials such as the prosthesis, little or no information about cement filling can be deduced. The uncertainty inherent in the resulting cement filling computation can be explicitly analysed. This uncertainty is essentially equal the degree that the result is affected by noise in the input image. Where dense metal objects like the prosthesis occlude the image, the useful signal is attenuated, while image noise remains constant. This results in a very low signal-to-noise ratio, resulting in unreliable cement filling estimates. For each pixel, the sensitivity of the filling fraction *F* to noise can be approximated as its derivative with respect to the observed fluoroscopic image intensity:

$$\begin{aligned} S= & {} \frac{\partial F}{\partial I_\mathrm{observed}}\\= & {} \frac{\partial }{\partial I_\mathrm{observed}}\frac{\log \left( I_\mathrm{observed}/I_\mathrm{none}\right) }{\log \left( I_\mathrm{full}/I_\mathrm{none}\right) }\\= & {} \frac{1}{I_\mathrm{observed}\log \left( I_\mathrm{full}/I_\mathrm{none}\right) }. \end{aligned}$$

*S* is only defined for the regions where *F* is directly computable. The reciprocal of the sensitivity function defines what we call the “certainty image” *C* :

4 $$\begin{aligned} C=I_\mathrm{observed}\log \left( I_\mathrm{full}/I_\mathrm{none}\right) . \end{aligned}$$

All non-computable pixels in *C* are set to zero. An example of the certainty image is shown in Fig. 11.

As was the case for *F*, we may re-project *C* along the projection rays and visualize it in three dimensions on the cement filling feedback panel. *F* and *C* are then combined in a single bi-variate colour mapping shown in Fig. 7.

The sensitivity of the overall cement volume estimate $V_\mathrm{filled}$ may be computed by applying the weights *W* to each individual pixel’s filling value sensitivity *S* in the same way as it was done in Eq. 3:

5 $$\begin{aligned} S_\mathrm{filled}=\frac{\sum _{x,y}\left( W_{x,y}\cdot S_{x,y}\right) }{\sum _{x,y}W_{x,y}}\cdot V_\mathrm{target}. \end{aligned}$$

## Experiments

Substituting real fluoroscopic images with simulated DRRs allow for perfect image registration, as all the required images could be generated using the exact same projection parameters. However, to test our software in a physically representative workflow using realistic clinical hardware, we enacted minimally invasive hip refixation procedures on five ex-articulated cadaver legs. We followed the workflow shown in Fig. 2. An experienced orthopaedic surgeon was asked to perform the minimally invasive cement injection—a procedure with which he was familiar.

### Preparation of cadaver specimens 

Five ex-articulated cadaver legs were obtained. Each leg included the whole femoral region, from the superior to the inferior epiphysis. In order to simulate peri-prosthetic lesions that could be filled with cement, we used a similar approach as that used by Kraaij et al. [15]. Two experienced orthopaedic surgeons placed cemented polished Exeter stems (Stryker, Limerick, Ireland) in each femur. Exeter stems of sizes 3 and 4 (both with an offset of 44 mm) were used, with the most appropriate size chosen for each leg.

Since we used cadaveric legs which were exarticulated in the hip joint, they provided free access to the femoral head and neck. The soft tissues of each legs were kept intact—this was important so as to provide a realistic target for percutaneous cement injection.

After each prosthesis was placed and the cement hardened, the prosthesis was again removed. The straight-edged and smoothly polished wedge-shaped Exeter prosthesis’ surface enables such removal without damaging the cement mantle [17]. After removing the prosthesis, a single cut through the skin and muscle tissue onto the bone surface was made with a dissecting knife, perpendicularly to the femur shaft. The femur itself was then sawed through with a bone saw. The leg was then tilted open along the cut line to allow access to the femur shaft where it was bisected. Lesions were created using an abrasive drill bit in an identical way as previously performed by Malan et al. [17]. The muscle tissue on the opposite side of the incision was kept intact, preventing the two semi-bisected halves of the leg to separate completely. After lesions were created, the prostheses were re-inserted. The snug fit between prosthesis and cement mantle ensured that the original alignment of the two partially bisected leg halves was restored in each case. The soft tissue around the incision perimeter was sewn closed so that the soft tissue could recover some degree of conformity as well.

### Pre-operative planning 

After preparation, each femur was CT scanned with its prosthesis in place. This CT image corresponded to the diagnostic pre-operative CT scan that a patient would routinely have performed if he/she was eligible for minimally invasive hip prosthesis refixation [8, 9].

The periprosthetic lesions of each leg were manually segmented in 3D from the CT volume using the Medical Imaging Interaction Toolkit (MITK 0.12.2), an interactive medical image segmentation software tool [20]. These segmented lesions defined the cement injection targets that we wished to fill in the subsequent minimally invasive cement injection step.

Following the workflow described in Fig. 2, we used HipRFX to read the CT image volume and the manually delineated segmentations and then to virtually place vertebroplasty needles to reach these lesions. We manually chose applicable angles for orienting the C-arm fluoroscope, after which we saved DRR snapshots of the expected view, both before and after cement injection.

### Cement injection

An experienced orthopaedic surgeon, who has previously performed percutaneous cement injection in more than thirty patients, performed the procedure on each of the prepared cadaver legs. Standard clinical grade needles (Biomet VerteShark Access, 11Gx15cm), vertebroplasty cement (Biomet Bone Cement V) and mixing sets (Optivac Procedure Set) were used—all supplied by Biomet Europe BV (Dordrecht, the Netherlands). Intraoperative fluoroscopy was performed using a standard clinical C-arm fluoroscope (Philips BV Pulsera , Best, the Netherlands).

**Fig. 15:**
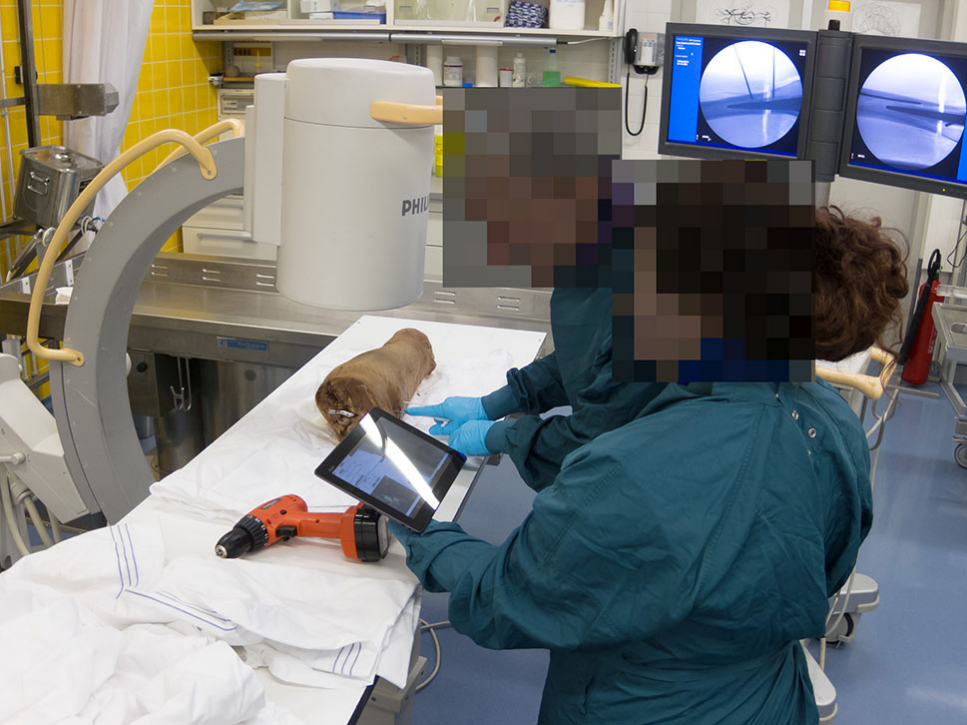
An assistant shows a simulated fluoroscopic image to the surgeon. Intraoperative fluoroscopic images are visible in the background. The cadaver leg is situated on the table, underneath the C-arm fluoroscope. The faces of the surgeon and assistant were anonymized for publication

As opposed to a traditional percutaneous procedure where no pre-operative guidance was available, an assistant held a 10.1-inch tablet computer (Asus TF700T, Taipei, Taiwan) in view of the surgeon on which the pre-computed DRR snapshots were displayed. A photograph of one of our experiments is shown in Fig. 15.

For each cadaver, the C-arm was moved to the appropriate position as indicated by the planning snapshot. Vertebroplasty needles were subsequently inserted as per pre-operative plan. As opposed to patients, the bones of the cadavers were not affected by osteolysis and necessitated insertion of the needles by first drilling into the bone using a thin drill bit. Even with pre-drilling, inserting the needles into the bone required a surgical hammer to supply sufficient force. In four of the five legs, both planned needles could be inserted, while needle breakage limited use to inserting a single needle in the remaining leg.

Polymethylmethacrylate (PMMA) bone cement was then mixed and injected under fluoroscopic C-arm guidance. The cement flow was fluoroscopically monitored in real time, as is usually done in this kind of procedure [8, 24]. Cement injection was continued until the periprosthetic space appeared filled or until the cement started leaking into the surrounding tissue.

### Acquisition of ground truth data

Fluoroscopic images obtained during our cadaver experiment were captured and saved to file. HipRFX was then set to simulate an equivalent projection viewpoint as used with the intraoperative C-arm fluoroscopy. Matching was performed manually, interactively and according to visual similarity, as the C-arm fluoroscope we used did not record the exact projection angles—neither visually nor in accompanying metadata. Our only guidelines to ensure consistency were the apparent agreement between the virtual C-arm positions and those used for the experiment, subjective agreement between the planning DRRs and those observed during the procedure, and the knowledge that HipRFX’s theoretical focal length and field of view matched those of the C-arm.

Two simulated DRRs were created for each real fluoroscopic image: one representing the pre-operative state, and a second representing ideal cement filling of the target lesions that were segmented by hand in the pre-operative CT volume. We used the procedure described in “Cement filling feedback panel” section to estimate cement filling.

After the cement had hardened, the cadaver legs were all returned to storage. A post-operative CT scan of each treated cadaver leg was performed. This step is not part of the workflow depicted in Fig. 2 but allowed for accurate and independent post-operative cement volume measurements to be performed, using manual delineation in the MITK software [19, 20].

**Table 1:** Cement filling estimates using HipRFX

Femur number	Target volume (ground truth) (ml)	Injected cement (incl. leaked) (ml)	Cement filling of target (ground truth) (%)	Filling estimate using HipRFX (%)	Cement volume estimation error as % of target (%)
1	3.58	11.64	51.8	46.2–50.3	5.6–1.6
2	3.20	8.90	52.0	46.8	5.2
3	3.17	3.75	33.4	31.2	2.3
4	4.64	4.69	17.6	32.5–21.8	14.9–4.2
5	4.09	5.03	55.4	65.3	9.8

Ground truth values were obtained by directly measuring them from independent post-operative CT volumes

## Results

**Fig. 16:**
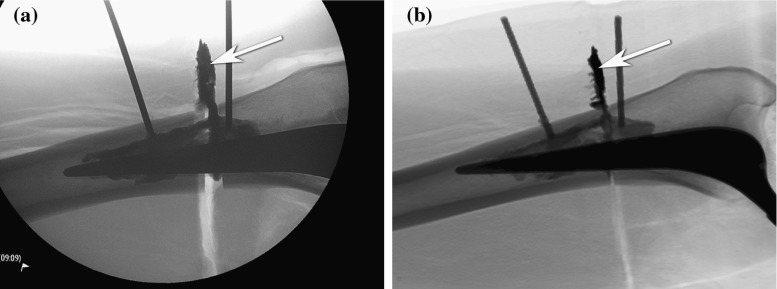
**a** Real fluoroscopy image during cadaver experiment on femur number 1—see Table 1. **b** HipRFX-generated DRR from post-operative CT with overlayed virtual needles. A region of cement leakage is indicated by *white arrows*. The incision created during preparation is visible as a light-coloured line extending into the zone of cement leakage

Figure 16 shows a side-by-side comparison between a fluoroscopy image obtained during the cadaver experiment and a corresponding HipRFX-generated DRR. Both of these images were generated using the actual experimental attained cement distribution—the fluoroscopic image created at the end of the cement injection, and the DRR generated using the post-operative CT volume. When the fluoroscopic image was made, the cement had not yet hardened. Some changes to the cement distribution are visible between the images, especially where cement leaked outside the femoral shaft. The physical incision that resulted from our experimental preparation method can be seen as the radiolucent band into which the cement leaked.

We observed that much of the leaked cement did so into the incisions created by our experimental method. This is clearly visible in Fig. 16. We compared all computed values with ground truth values measured in post-operative CT. With a median of 81 % (range 55–84 %), the cement leakage in our experiment was higher than observed in real patients [18]. The filling percentage (median 52 %, range 18–55 %) was comparable to that observed in real patients [18]. The properties of the experimentally created cadaver leg lesions and the subsequent injected cement volumes are shown in Table 1.

For each real fluoroscopic image, HipRFX was used to estimate the amount of cement that was injected into the target region. Two of the five legs were each imaged from two different angles. The median estimation error, expressed as a percentage of the cement target volumes, was 5.2 % (range 1.6–14.9 %). These results are also summarized in Table 1.

**Fig. 17:**
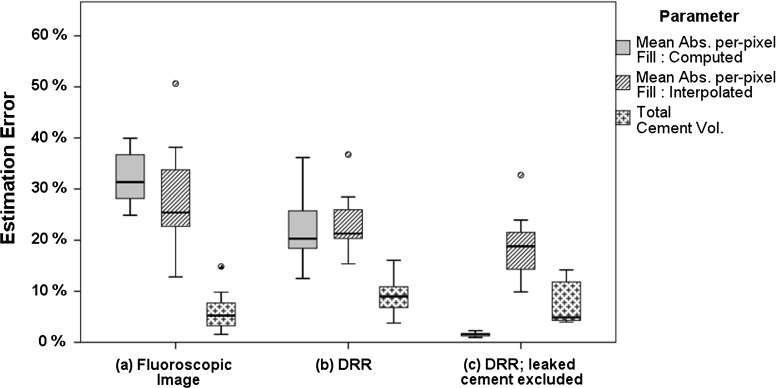
Experimentally obtained cement filling estimation errors using HipRFX. **a** Errors when analysing real fluoroscopic data compared to **b** the equivalent errors when substituting real fluoroscopic images with simulated fluoroscopic images. In **c**, cement leakage was omitted from the simulated images. Mean absolute per-pixel errors as well as the final integrated cement volume estimate errors are shown

To examine the role that experimental inaccuracies and assumptions had on our results, we further differentiated the absolute per-pixel errors made in the 2D “filling images” like those in Fig. 11. We compared results when using either (a) our real experimentally obtained fluoroscopic images that include unaccounted-for cement leakage, image noise and possible geometric-and-image-intensity registration errors, (b) simulated fluoroscopic images that include unaccounted-for cement leakage but with perfect registration and (c) simulated fluoroscopic images with no cement leakage. For each of these scenarios, we furthermore distinguished between the estimation error made in the “computable” and the “non-computable” interpolated regions that are described and illustrated in Fig. 14. Results are shown in Fig. 17.

The simulated fluoroscopic images for (b) were DRRs that used the complete post-operative 3D cement distribution, segmented in the post-operative CTs. The simulated fluoroscopic images for (c) were identical to (b) except that it excluded all leaked cement. As our cement filling algorithm does not model cement leakage, this modification meets the assumption inherent in our cement estimation algorithm, which is that all injected cement projecting onto the image of the cement target must also be located within the cement target.

After performing the experiment, we discussed our proof-of-concept software with the orthopaedic surgeon who participated. He valued the pre-operative planning capabilities that the software offers without necessitating additional or new clinical hardware. He stated that HipRFX represented a promising development in image-based guidance and also represents a step towards reducing the need for intraoperative CT imaging.

## Discussion

Minimally invasive hip refixation by cement injection is a novel and experimental technique for refixing loosened orthopaedic implants [8, 24]. To our knowledge, there currently exists no task-specific tool for planning or performing this procedure. Our experimental HipRFX system has been purpose-designed for this task and uses existing fluoroscopic hardware to provide intraoperative guidance and to analyse the distribution of injected cement. HipRFX can simulate fluoroscopic images and computes cement volume estimates and a combined filling-and-uncertainty distribution. Should it be used intraoperatively, our system could provide a surgeon with valuable extra information that currently is either absent, or can only be obtained after the fact. HipRFX focuses on simulating and analysing the fluoroscopic images a surgeon would see—including the realistic fluoroscopic appearance of injected bone cement. This allows us to progress beyond needle guidance and to also simulate and assess the cement filling.

By visually judging the agreement between a simulated desired outcome and real intraoperative images, a surgeon could be aided in judging whether sufficient cement penetration has been achieved. An obvious deviation between simulated and observed fluoroscopic images may alert him to unwanted outcomes, such as cement leakage. In addition, we experimentally showed that HipRFX can compute numerical estimates of the injected cement volume.

Our pre-operative software tool is built around the assumption that C-arm fluoroscopy is the dominant intraoperative technology for monitoring percutaneous cement injection and will most likely remain so for the foreseeable future. Additionally, we assume that at least a single pre-operative CT image volume will be available before commencement of surgical planning. These two assumptions are compatible with existing radiological protocol for minimally invasive cement injections [8, 24].

The Philips XperGuide [10, 16] system shows some similarities with our approach to intraoperative guidance as it also bases its guidance on a prior CT volume and also combines this with live single-plane C-arm fluoroscopy. However, XperGuide is focused on guiding needle biopsies only and achieves this by overlaying glyphs onto radiological images. Unlike XperGuide, our system is, in principle, compatible with all existing CT imagers and fluoroscopes. This was demonstrated in our cadaver experiment where we used a commercial C-arm fluoroscope that precluded any direct calibration between our software and the imaging hardware. Here we showed that HipRFX is capable of estimating the volume of cement to have reached pre-operatively defined target lesions. While we used post-operative CT as ground truth to compare our results to, post-operative CT does not form any part of our workflow as shown in Fig. 2.

Where they project to the same pixel positions, our algorithm cannot distinguish leaked cement from cement inside the target region. A large amount of leaked cement may therefore negatively affect the accuracy of the cement filling estimation, whereby the algorithm interprets leaked cement to be located in the target lesion. This view is supported by Fig. 17 that shows much reduced estimation errors when we analysed DRRs where leaked cement was digitally removed.

An independent analysis of real patient data showed that cement leakage is prevalent in clinical practice [18]. Given this reality of cement leakage, HipRFX plays the important role of specifically estimating the injected cement fraction that reaches a target lesion. This fraction is presumed to be the one that directly contributes to the stability of a hip prosthesis [2]. Cement leakage was exacerbated in our experiments by the cadaver legs having been sawed through, thereby providing an easy escape route for injected cement. Even in this challenging scenario, the median cement volume estimate error was 5.2 % (range 1.6–14.9 %). We are encouraged by this result, especially considering the limitations of our experimental set-up.

Residual image registration mismatches limited the accuracy with which our algorithm could deduce cement filling. From Fig. 17, we made the paradoxical observation that, for the fluoroscopy images, the estimation error was higher in computable than in interpolated regions. We explain this by random image noise being present in fluoroscopic images that cause per-pixel fluctuations in filling estimation. By contrast, the interpolated regions are smooth and show a lower per-pixel absolute error. This explanation is supported by the observation that the errors in the computable regions decreased when we substituted the fluoroscopic images with DRRs, while the interpolated regions’ error decreased by less. We observed that per-pixel estimation errors tended to cancel out when they were summed, resulting in a reduced estimation error for the total cement volume.

A limitation of HipRFX in its current form is its inability to directly record the position and projection parameters of the relevant fluoroscope. In fact, in our experiments, these parameters were not numerically recorded at all. The simulated C-arm was visually and manually aligned to match the DRRs with the recorded fluoroscopic image. The inevitable discrepancies that resulted from such qualitative alignment required us to perform image registration, as schematically illustrated in Fig. 2. Having to deal with fewer unknowns and the use of iterative techniques that better match radiographs with CT-derived DRRs [12, 26] may, in future, improve registration accuracy. Calibrated systems using accurate angle encoders such as the Philips XperGuide may, in future, be constructed for our application and make the system easier to use and more robust.

In clinical practice, needle placement is mainly performed under the supervision of single-plane fluoroscopy [8, 24]. Intraoperative CT may also be used for more accurate 3D guidance. Similar to Philips XperGuide, our software has the potential to be developed to guide a surgeon during needle placement. One possibility would be to simulate a correctly placed needle and overlay it in live fluoroscopy. We leave the exploration of this topic to future work.

Our system may, in future, be used to compute optimal C-arm orientations for performing an intervention. This can be done by maximizing the image difference between DRRs of the expected pre-operative and post-operative situation. Large image differences indicate that the changes caused by the planned procedure are clearly visible—this then represents an informative viewing angle. Differences may enable discernment between correct and incorrect needle placement, or between sufficient and insufficient cement filling. The parameter space that needs to be traversed in this case consists only of the C-arm’s elevation and azimuth—i.e. a two- dimensional optimization problem. When a C-arm fluoroscope needs to be positioned accurately to within $5{\circ }$, all possible orientations may be examined in fewer than 800 iterations. Given the speed with which DRRs and difference images can be computed, this brute force approach would be feasible for optimizing the view for each desired step in the cement injection procedure, while being guaranteed to find the global optimum.

Another direction for future work would be to combine the estimates obtained from several distinctly oriented fluoroscopy images. A stereo fluoroscopy system [3] may be used for this purpose, or several single-plane fluoroscopic images may be taken successively from different angles. The area occluded by a large metal prosthesis would be different for each image and the combined estimate may be considerably more accurate than either estimate on its own.

We are encouraged by the feedback received from the orthopaedic surgeon who used HipRFX in planning and executing the cadaver experiment. We foresee that, in future, the techniques described in this paper could provide surgeons with sufficient insight and feedback on percutaneous cement injection procedures to avoid the use of explicit 3D imaging tools in the operating room. We believe that the ability to glean quantitative estimates of the cement volume that is deposited in a desired target region, without having to resort to 3D imaging modalities, is novel. This approach may be applied to any application where radiopaque cement in injected, including vertebroplasty.
